# Total marrow lymphoid irradiation IMRT treatment using a novel CT-linac

**DOI:** 10.1186/s40001-023-01380-4

**Published:** 2023-10-27

**Authors:** Dazhen Jiang, Di Deng, Yu Xiong, Dajiang Wang, Jian Gong, Hongli Zhao, Zhirong Bao, Yongchang Wei, Conghua Xie, Lecheng Jia, Can Liao, Shuo Liu, Hui Liu, Xiaoyong Wang

**Affiliations:** 1 Department of Radiation and Medical Oncology, Hubei Key Laboratory of Tumor Biological Behaviors, Hubei Cancer Clinical Study Center, Zhongnan Hospital of Wuhan University, Wuhan, 430071 China; 2 United Imaging Research Institute of Innovative Medical Equipment, Shenzhen, 518045 China; 3 Shanghai United Imaging Healthcare Co., Ltd, Shanghai, 201807 China

**Keywords:** Total Marrow and Lymphoid Irradiation, CT-linac, Radiotherapy, Bone marrow transplantation

## Abstract

**Background:**

A novel CT-linac (kilovolt fan-beam CT-linac) has been introduced into total marrow and lymphoid irradiation (TMLI) treatment. Its integrated kilovolt fan-beam CT (kV FBCT) can be used not only for image guidance (IGRT) but also to re-calculate the dose.

**Purpose:**

This study reported our clinical routine on performing TMIL treatment on the CT-linac, as well as dose distribution comparison between planned and re-calculated based on IGRT FBCT image sets.

**Methods:**

11 sets of data from 5 male and 6 female patients who had underwent the TMLI treatment with uRT-linac 506c were selected for this study. The planning target volumes consist of all skeletal bones exclusion of the mandible and lymphatic sanctuary sites. A planned dose of 10 Gy was prescribed to all skeletal bones exclusion of the mandible in two fractions and 12 Gy in two fractions was prescribed to lymphatic sanctuary sites. Each TMLI plan contained two sub-plans, one dynamic IMRT for the upper body and the other VMAT for the lower extremity. Two attempts were made to obtain homogeneous dose in the overlapping region, i.e., applying two plans with different isocenters for the treatment of two fractions, and using a dose gradient matching scheme. The CT scans, including planning CT and IGRT FBCT, were stitched to a whole body CT scan for dose distribution evaluation.

**Results:**

The average beam-on time of Planupper is 30.6 min, ranging from 24.9 to 37.5 min, and the average beam-on time of Planlower is 6.3 min, ranging from 5.7 to 8.2 min. For the planned dose distribution, the 94.79% of the PTVbone is covered by the prescription dose of 10 Gy (V10), and the 94.68% of the PTVlymph is covered by the prescription dose of 12 Gy (V12). For the re-calculated dose distribution, the 92.17% of the PTVbone is covered by the prescription dose of 10 Gy (V10), and the 90.07% of the PTVlymph is covered by the prescription dose of 12 Gy (V12). The results showed that there is a significant difference (*p* < 0.05) between planning V10, V12 and delivery V10, V12. There is no significant difference (*p* > 0.05) between planned dose and re-calculated dose on selected organs, except for right lens (*p* < 0.05, Dmax). The actual delivered maximum dose of right lens is apparently larger than the planned dose of it.

**Conclusion:**

TMLI treatment can be performed on the CT-linac with clinical acceptable quality and high efficiency. Evaluation of the recalculated dose on IGRT FBCT suggests the treatment was delivered with adequate target coverage.

## Introduction

In recent years, total marrow irradiation (TMI) and total marrow/lymphoid irradiation (TMLI) are being performed in many institutions [1–14], as conditioning regimens prior to hematopoietic stem cell transplantation (HCT). Compare to conventional total body irradiation (TBI), TMI/TMLI have been shown to be superior in reducing organ toxicities, which target bone and the major lymph node chains while sparing critical organs, e.g., kidneys, lungs, and liver. Due to the large complex target shapes of TMI/TMLI, a suitable device is required for TMI/TMLI delivery. Helical Tomotherapy (HT) is a radiation therapy delivery device that allows for an image-guided intensity-modulated radiation therapy treatment (IMRT) of the target up to 135 cm, making it appropriate for such treatments. The feasibility and clinical data of HT-based TMI/TMLI have been reported by several previous studies [9–14], showing the potential of HT-based TMLI for dose escalation with acceptable toxicity. In [9], our institution also implemented the TMI/TMLI in clinical practice with HT system, demonstrating the technical feasibility of HT-based TMI/TMLI using the total prescription dose of 8–10 Gy delivered by 2 fractions within one day. Although HT-based TMI/TMLI have achieved these encouraging results, they also have some limitations, such as long treatment time and limited availability of HT system (Accuray Inc., Sunnyvale, CA). Hence, some researchers also attempted to develop the clinical acceptable TMI/TMLI plans, which can be delivered by conventional C-arm linear accelerators [1–8]. For example, Han [1] evaluated the feasibility of adopting volumetric modulated arc therapy (VMAT) to deliver TMI treatment, and the results showed that VMAT-based treatment on conventional C-arm linac can achieve a comparable plan quality while showing significant reduction on beam-on time, compared with HT-based treatment. All these existing studies (HT-based or C-arm linac based) focused on the dose distribution evaluation of the treatment plan. However, the true dose distribution of delivery is also important in clinical practice.

The uRT-linac 506c linear accelerator (United imaging HealthCare co., LTD, Shanghai, China) is a unique device that combines diagnostic kilovolt CT with high dose rate intensity modulated accelerator, making it capable of performing precise radiotherapy with high resolution CT image guided, implementing online adaptive radiotherapy, evaluating the dose distribution of delivery, and so on [15, 16]. Compared with tomotherapy and traditional linac, uRT-linac 506c linear accelerator can not only effectively complete the treatment of TMI/TMLI patients with upper or lower body, but also verify the actual dose transmitted to patients based on diagnostic fan beam CT. In this paper, we aim at investigating the technical feasibility of the uRT-linac 506c for TMLI and evaluating the actual dose distribution of delivery with uRT-linac 506c. To the best of our knowledge, this is the first research that re-calculate and evaluate dose distribution on IGRT images for TMI/TMLI treatment.

## Materials and methods

### Patient characteristics and CT simulation

From October 2020 to March 2021, eleven participants who underwent TMLI treatments with uRT-linac 506c linear accelerator were included for retrospective study. The mean age of participants is 28 years (range from 13 to 49 years) of whom 5 are male and 6 are female. This study was approved by the medical ethics committee Zhongnan Hospital of Wuhan University.

Because of the limitation of treatment couches and scanning length of CT, two simulation CT scans are required to cover the whole body of patient. Note that we define these two scans as the upper scan (CT_upper_) and the lower scan (CT_lower_) in this paper. The upper body scan is acquired in the head first supine (HFS) orientation, while the lower limb scan is acquired in the feet first supine (FFS) orientation. For the treatment purpose, the patients were originally scanned with a 16 slice CT simulator (Sensation Cardiac 64x, Siemens, Munich, Bavaria, Germany) using 65 cm field of view, 512*512pixels, and 5 mm slice thickness. All the planning CT scans were performed in the shallow normal-breathing mode. Target definition and dose fractionation can be found in our previous article [9].

**Fig. 1:**
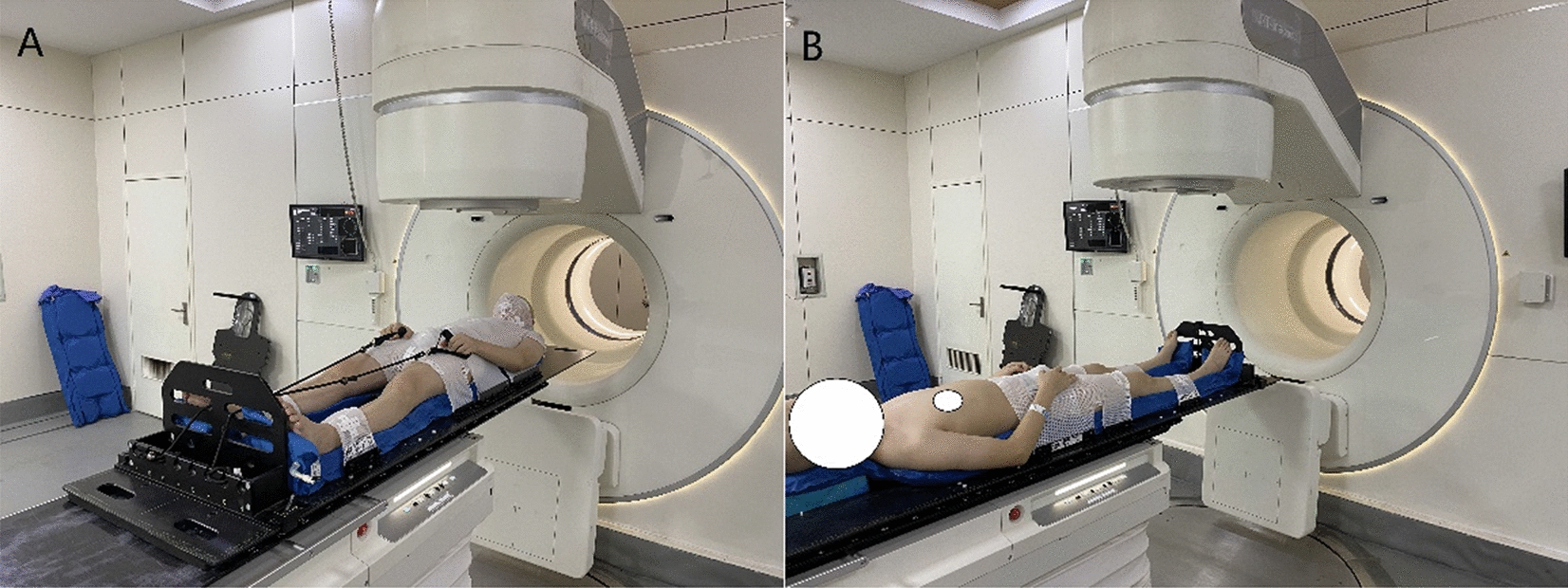
Examples of immobilization system used in this paper. **A** head first supine (HFS) position; **B** feet first supine (FFS) position

Figure 1 shows the patient immobilization devices used in our study, which include one the body frame, one whole body vacuum bag, one upper limb fixator, and three thermoplastic masks. Specifically, to reduce the interfraction and intrafraction motion of the upper limbs during a course of treatment, patients are fixed with fingers grasping the rope. The details of immobilization approach were thoroughly described in our previous study [9].

### Treatment planning

**Fig. 2:**
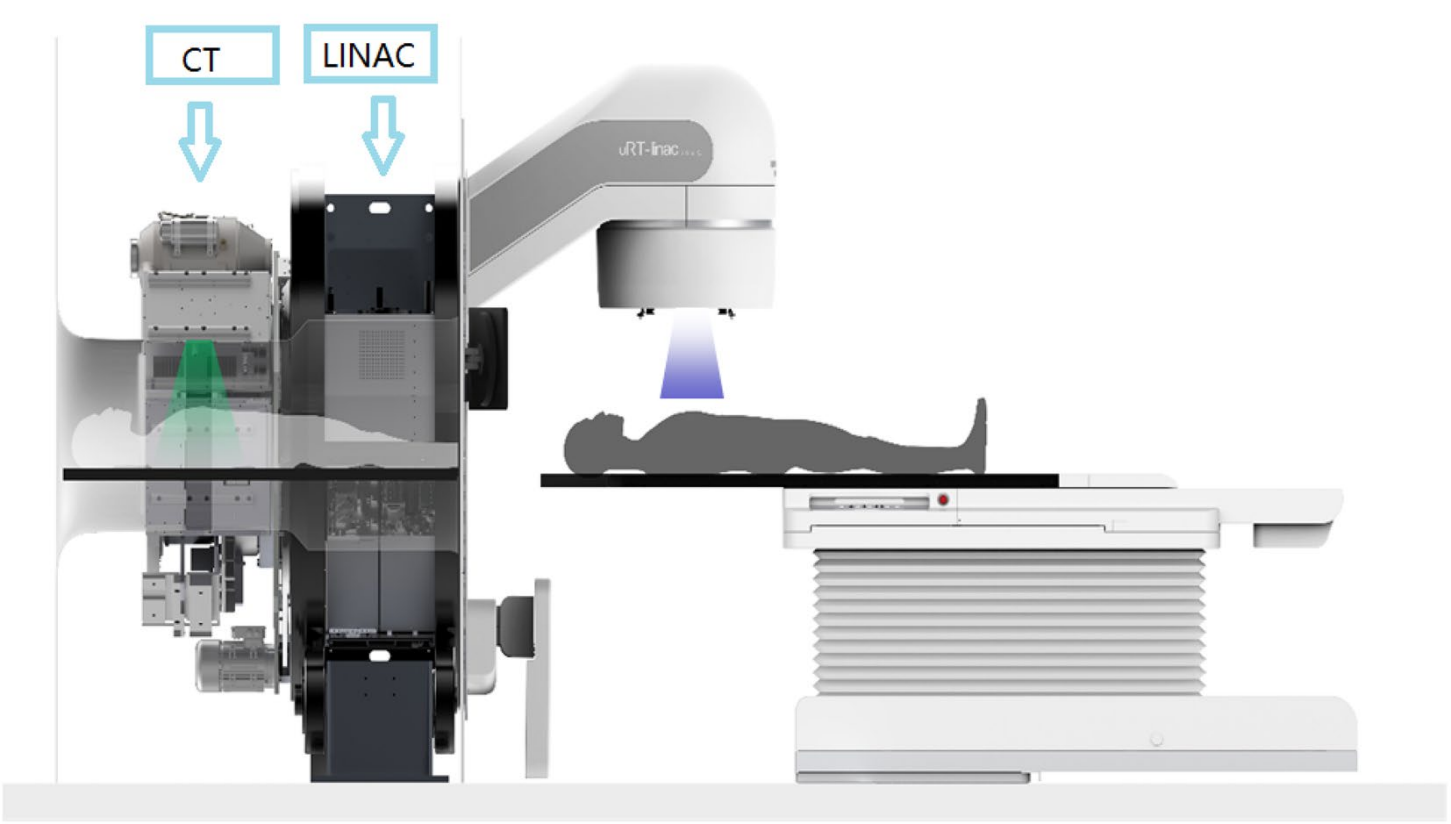
The linear accelerator of United Imaging Healthcare's CT linac URT-Linac 506C

Main characteristics of the uRT-linac 506c are as following: C-arm linear accelerator equipped with a 16-slice helical CT scanner, generates and delivers photon beams of two energies, i.e., the 6-MV treatment beam and 1.5-MV imaging beam as we can see Fig. 2. The 6-MV treatment beam can be delivered in flattened and unflattened modes with a maximum dose rate of 600 and 1400 MU/min, respectively. The treatment head is equipped with two pairs of collimating jaws and 60 pairs of MLCs with a 0.5-cm width at the isocenter in the inner 20 cm and a 1.0-cm width in the outer 20 cm, projecting a maximum field size of 40 cm × 40 cm. The available delivery techniques are three-dimensional conformal radiation therapy (3D-CRT), step-and-shoot IMRT (sIMRT), dynamic IMRT (dIMRT) and volumetric modulated arc therapy (VMAT). The gantry of the linac enables one and a half continuous rotation from − 362° to 182°, with a maximal rotation speed of 7°/s. The treatment couch has four-degrees-of-freedom (4DOF), allowing lateral, longitudinal, vertical translations and yaw rotation.

Treatment plans were optimized on uTPS (R001) which is a treatment planning system developed by United imaging HealthCare (UIH) Company. Because of the limitation of maximal longitudinal treatment length, two plans (Plan_upper_ and Plan_lower_) were designed for treatment of one patient, where Plan_upper_ was designed for upper body target and Plan_lower_ was designed for lower extremity target. Specifically, Plan_upper_ was performed by dIMRT with 4 isocenters located on head, chest, abdomen, and pelvis. 5–7 beams were selected at each isocenter. Plan_lower_ was performed by VMAT technique with 1 full arc at each of the 3 isocenters.

In radiation oncology, TMI/TMLI plan is one of the most complex plans owing to complicacy and extent of the targets, and the number of involved organs at risk. To avoid hotspots and coldspots in the junction region of CT_upper_ and CT_lower_, we applied a dose gradient matching scheme. The dose distribution in last few slices from cranial–caudal direction of CT_upper_ was optimized to obtain a dose gradient going from 100 to 20% of the prescribed dose in 40 mm. For this purpose, we assigned 80%, 60%, 40%, 20% of the prescribed dose to the target on the last few slices, respectively (four dose control rings were contoured on the top of the thigh and each ring includes one or two slices). Like the same, the dose distribution in the first several slices from caudal direction of CT_lower_ was optimized to acquire a dose gradient going from 20 to 100% of the prescribed dose in 40 mm, so that the dose distribution of junction region in Plan_upper_ can be complemented. In this way, an integral homogenous dose distribution was obtained in the junction region. We first combined four dose control rings and then extended one slice to generate an overlap region, which was defined as PTV_junction_ in this paper.

All plans were generated adopting an identical set of PTV/OAR dose-volume constraints. The criterion for acceptance of the plan was that at least 90% of the PTV received the prescription dose, with the normal organ dose to a minimum. This criterion for the prescription dose of the PTV is more restrict than the previous studies in which the TMI/TMLI treatment requires that at least 85% of the PTV volume achieved the prescription dose [15].

### Treatment delivery

The first step of treatment delivery process is patient setup, aligning patient markers to the laser. For the treatment of upper body, the patients were positioned in the head first supine orientation (HFS). For the treatment of lower extremity, the patients were positioned in the feet first supine orientation (FFS). Then the couch was shifted automatically to deliver the treatment of each isocenter. Image-guided radiotherapy (IGRT) was performed with the fan beam CT (FBCT) integrated in uRT-linac 506c at selected isocenters prior to the treatment to verify the patient position. Correction to patient position can be applied if the IGRT offset exceeds tolerance 3 mm in our institution [9]. To balance the treatment accuracy and efficiency, IGRT was performed at all isocenters of Plan_upper_ and the two isocenter of the Plan_lower_. The length of the IGRT scans was selected to make sure the knee and the ankle covered.

### Treatment evaluation

**Fig. 3:**
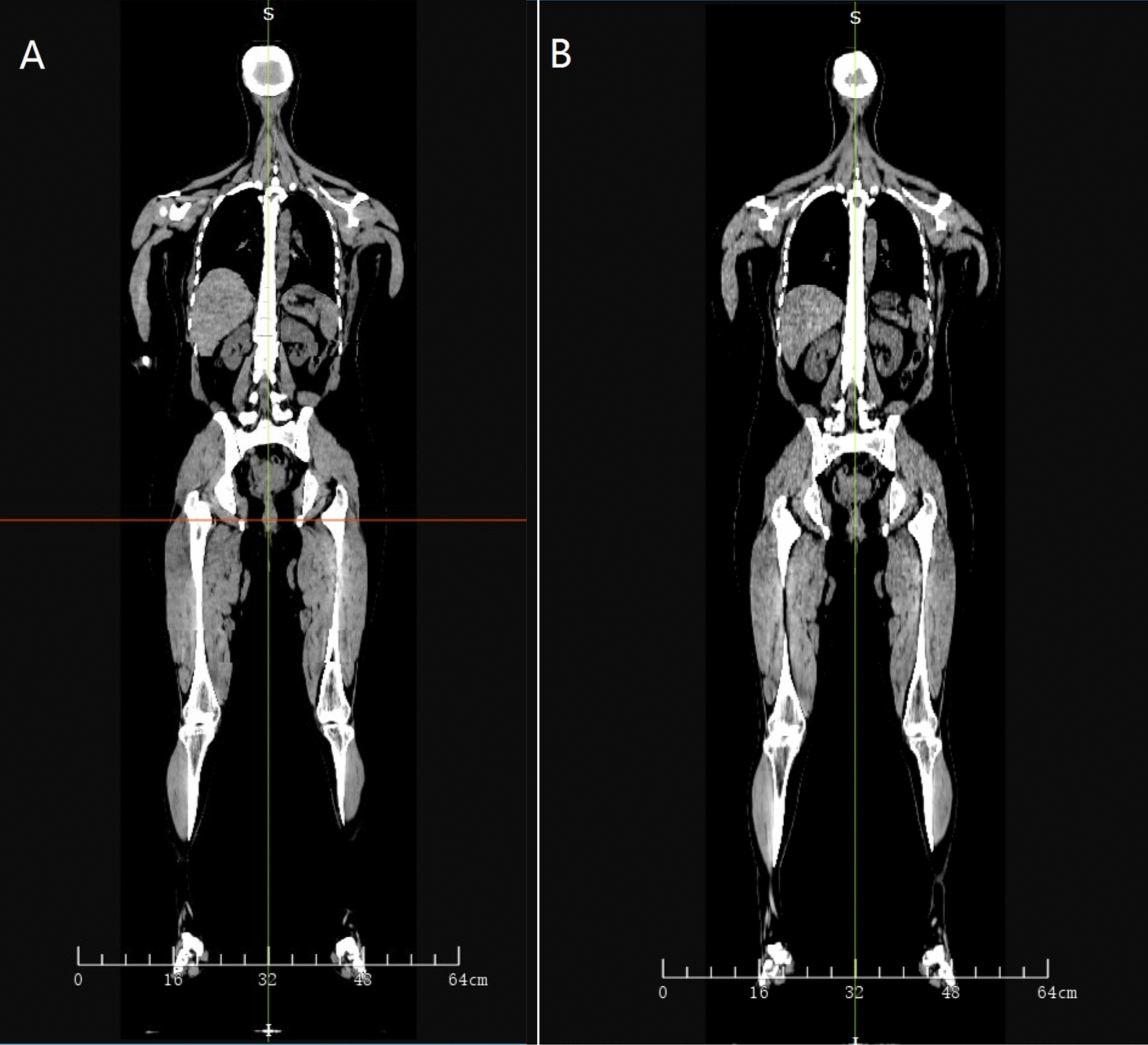
Examples of the whole body CT. **A** Planning CT. **B** FBCT scanned in the first fraction

In this study, we calculated the dose distribution on the whole body planning CT as well as on the whole body FBCT. Specifically, FBCTs at head, chest, abdomen, pelvis, and lower limbs were obtained for the purpose of IGRT originally. All the whole body CT (including planning CT and FBCT) were stitched with an image analysis tool provided by UIH company. Figure 3 shows the examples of the whole body CT. The whole body planning CT was generated by rigid registration of upper body CT and lower body CT. The lower body CT was adopted for the overlapped part. As for FBCT stitching, they were firstly registered to the whole body planning CT with the rigid registration matrixes obtained during IGRT. As for the overlap parts between FBCTs, the scan of the lower part of the body was adopted as well. To obtain the dose distribution on the whole body CT, we first used uTPS to duplicate the Plan_upper_ and Plan_lower_ to the planning CT and FBCT of the whole body, and then utilized uTPS to calculate the dose distribution. In this paper, the dose distribution calculated on the whole body planning CT is defined as the planned doseplanned dose distribution and the dose distribution calculated on the whole body FBCT is defined as the re-calculated dose distribution. The re-calculated dose is used to approximate the delivered dose to the patient.

### Statistical analysis.

Data were analyzed using the Statistical Package for Social Sciences version 22.0 (IBM Corporation, Armonk, NY, USA). The significance of differences between mean values was determined by Wilcoxon signed-rank test. P-values < 0.05 were considered statistically significant.

## Results

### Treatment parameters

The beam-on time for upper and lower body treatments over 11 patients are shown in Table 1. The average beam-on time of Plan_upper_ is 30.6 min, ranging from 24.9 to 37.5 min, and the average beam-on time of Plan_lower_ is 6.3 min, ranging from 5.7 to 8.2 min. In addition, the total treatment time is between 1 and 1.5 h, comprising patient alignment, IGRT, position reversal (HFS to FFS), etc.

**Table 1:** The beam-on time for upper and lower body treatments over the 11 patients

The beam on time (min)	Mean	Range
Plan_upper_	30.6	24.9–37.5
Plan_lower_	6.3	5.7–8.2
Plan _upper_ + Plan _lower_	36.9	31.3–41.0

### Dosimetry

Table 2 shows the quantitative DVH analysis of planned doseplanned dose and re-calculated dose for PTV. Note that we applied the prescription dose of 10 Gy to PTV_bone_ and the prescription dose of 12 Gy to PTV_lymph_, For the planned doseplanned dose distribution, the 94.79% of the PTV_bone_ is covered by the prescription dose of 10 Gy (V_10_), and the 94.68% of the PTV_lymph_ is covered by the prescription dose of 12 Gy (V_12_). For the re-calculated dose distribution, the 92.17% of the PTV_bone_ is covered by the prescription dose of 10 Gy (V_10_), and the 90.07% of the PTV_lymph_ is covered by the prescription dose of 12 Gy (V_12_). We conducted Wilcoxon signed-rank test between planning V_10_, V_12_ and delivery V_10_, V_12_. Although the results showed that there is a significant difference (p < 0.05) between planning V_10_, V_12_ and delivery V_10_, V_12._ Where V_10_ in PTV_bone_ and V_12_ in PTV_lymph_ were larger than 90%.

**Table 2:** DVH analysis of planned dose and re-calculated dose for PTV

PTV		Dmean (Gy)	Dmax (Gy)	D_95_ (Gy)	D_90_ (Gy)	V_7_ (%)	V_8_ (%)	V_9_ (%)	V_10_ (%)	V_11_ (%)	V_12_ (%)
**PTV**_**bone**_ (10 Gy)	Plan	11.31 $\pm$ 0.30	14.75 $\pm$ 0.58	9.98 $\pm$ 0.21	10.36 $\pm$ 0.17	99.57 $\pm$ 0.23	99.23 $\pm$ 0.26	98.39 $\pm$ 0.62	94.79 $\pm$ 1.59	65.85 $\pm$ 10.39	20.86 $\pm$ 13.31
	re-calculated	11.18 $\pm$ 0.27	14.96 $\pm$ 0.76	9.55 $\pm$ 0.23	10.20 $\pm$ 0.14	98.53 $\pm$ 0.50	97.87 $\pm$ 0.53	96.55 $\pm$ 0.64	92.17 $\pm$ 1.35	64.56 $\pm$ 10.89	23.72 $\pm$ 16.66
**PTV**_**lymph**_ (12 Gy)	Plan	12.65 ± 0.16	14.36 ± 0.65	11.92 ± 0.23	12.22 ± 0.10	99.68 ± 0.24	99.53 ± 0.35	99.30 ± 0.51	98.93 ± 0.73	98.20 ± 1.09	94.68 $\pm$ 1.97
	Re-calculated	12.50 ± 0.19	14.38 ± 0.66	11.10 ± 1.24	11.96 ± 0.27	99.14 ± 1.06	98.73 ± 1.55	98.31 ± 1.72	97.65 ± 1.89	96.26 ± 2.24	90.07 $\pm$ 5.13

Table 3 summarizes the dosimetric data of the organs at risk (OAR). Wilcoxon signed-rank test was conducted on D_mean_ and D_max_ between the plan and the delivery of the selected OARs. We found that there is no significant difference (p > 0.05) between planned dose and re-calculated dose on most of selected organs, except for right lens (p < 0.05, D_max_). The actual delivered maximum dose of right lens is apparently larger than the planned dose of it. This difference of planned dose and re-calculated dose comes from the poor repeatability of the patient position. We will thoroughly discuss it in Sect. "Discussion". In summary, the consistency of planning and re-calculated dose distribution can reflect the good treatment quality to some extent.

**Table 3:** DVH analysis of planned dose and re-calculated dose for organ at risk (OAR)

		Dmean (Gy)	Dmax (Gy)	D_50_ (Gy)	V_2_(%)	V_4_(%)	V_6_ (%)	V_8_ (%)
Small bowel	Plan	5.88 ± 0.95	12.26 ± 1.33	5.22 ± 0.88	100 ± 0	80.14 ± 10.10	35.84 ± 14.71	19.79 ± 13.43
	Re-calculated	5.89 ± 1.01	12.53 ± 1.18	5.37 ± 1.14	100 ± 0	80.06 ± 10.40	35.89 ± 15.21	19.57 ± 14.36
Heart	Plan	5.80 ± 1.07	12.68 ± 1.42	4.91 ± 0.77	99.83 ± 0.41	72.94 ± 13.93	33.96 ± 12.19	18.58 ± 13.01
	Re-calculated	5.81 ± 1.26	12.13 ± 2.02	5.00 ± 0.87	99.67 ± 0.93	71.10 ± 15.04	34.18 ± 14.74	19.11 ± 15.31
Left Kidney	Plan	3.77 ± 0.42	10.24 ± 1.80	3.30 ± 0.40	99.72 ± 0.87	30.61 ± 10.56	8.17 ± 5.36	1.84 ± 2.61
	Re-calculated	4.17 ± 0.89	10.53 ± 2.19	3.55 ± 0.58	99.68 ± 1.02	36.02 ± 14.52	13.90 ± 12.32	5.98 ± 8.74
Right Kidney	Plan	3.60 ± 0.32	9.83 ± 1.90	3.19 ± 0.28	98.61 ± 2.92	26.73 ± 8.71	6.61 ± 4.82	1.29 ± 1.85
	Re-calculated	3.84 ± 0.63	10.32 ± 1.86	3.29 ± 0.44	98.58 ± 3.13	30.74 ± 13.79	11.37 ± 8.40	4.25 ± 4.98
Left Lens	Plan	1.40 ± 0.26	1.51 ± 0.31	1.39 ± 0.26	0.00	0.00	0.00	0.00
	Re-calculated	1.54 ± 0.52	1.99 ± 0.84	1.50 ± 0.49	16.31 ± 24.71	0.00	0.00	0.00
Right Lens	Plan	1.41 ± 0.24	1.52 ± 0.29*	1.41 ± 0.24	0.00	0.00	0.00	0.00
	Re-calculated	1.51 ± 0.41	1.85 ± 0.63*	1.50 ± 0.38	10.45 ± 22.80	0.00	0.00	0.00
Liver	Plan	5.47 ± 0.89	12.47 ± 1.24	4.76 ± 0.70	99.37 ± 1.08	61.94 ± 11.79	34.49 ± 11.48	18.69 ± 12.51
	Re-calculated	5.53 ± 0.97	12.64 ± 1.20	4.85 ± 0.85	99.02 ± 1.39	62.22 ± 13.11	35.03 ± 13.69	20.41 ± 12.54
Left Lung	Plan	5.58 ± 0.50	13.23 ± 0.56	5.03 ± 0.47	99.71 ± 0.90	64.93 ± 7.21	38.54 ± 6.28	19.17 ± 5.89
	Re-calculated	5.53 ± 0.53	13.06 ± 0.55	4.94 ± 0.46	99.59 ± 1.01	63.33 ± 5.80	37.50 ± 6.27	18.85 ± 7.03
Right Lung	Plan	5.52 ± 0.44	13.06 ± 0.75	5.01 ± 0.43	99.27 ± 1.49	64.52 ± 6.53	38.66 ± 5.57	19.03 ± 5.36
	Re-calculated	5.42 ± 0.44	12.86 ± 0.66	4.87 ± 0.38	99.25 ± 1.53	63.25 ± 6.14	36.73 ± 5.05	17.52 ± 5.52
Stomach	Plan	7.28 ± 1.45	12.88 ± 0.82	7.18 ± 1.59	100	94.59 ± 5.56	66.01 ± 19.74	35.98 ± 29.31
	Delivery	7.21 ± 1.65	12.49 ± 1.19	7.13 ± 1.73	100	91.35 ± 10.39	63.62 ± 24.20	37.84 ± 31.09

* Means that there is significant difference (p < 0.05) between planned dose and re-calculated dose on selected organs

### Dose evaluation in the overlapping area

Table 4 lists the received dose on the PTV_junction_ (defined in Sec.2.3) over 11 patients. The prescription dose of PTV_junction_ is 10 Gy. For planning, 90% of the PTV_junction_ was covered by the prescription dose. The average dose (11.1 Gy) and the maximum dose (13.4 Gy) in the PTV_junction_ were greater than the prescription dose (10 Gy), which is similar to those reported in previous studies [9]. In addition, as we mentioned in 2.3, a dose gradient matching scheme was applied in the planning stage to reduce hotspots in the junction region. Here, the maximum dose in the PTV_junction_ was 134% of the prescription dose for plan, which is lower than the previous study [9] (the maximum dose in the junction region was 140% of the prescription dose for plan). For delivery, 90% of the PTV_junction_ was covered by the prescription dose and the maximum dose in the PTV_junction_ was 132% of the prescription dose. We cannot find a study that evaluated the dose distribution of delivery. We conducted wilcoxon signed-rank test between the planned dose and re-calculated dose (including D_95_, D_90_, mean, and max), and the results showed that there is no significant difference between them.

**Table 4:** The received dose on the PTV_junction_ over 11 patients

	D_95_(Gy)	D_90_(Gy)	Mean	Max
Plan	10.0 ± 0.72	10.3 ± 0.70	11.1 ± 0.62	13.4 ± 0.83
Delivery	9.82 ± 0.77	10.3 ± 0.61	11.1 ± 0.57	13.2 ± 0.75

## Discussion

In recent years, the total marrow irradiation gradually replaced the total body irradiation in acute myeloma and leukaemia, which can reduce the toxicity of irradiation. At present, the complexity of TMLI plan and long irradiation time may cause the dose distribution difference between plan and delivery. A novel CT-linac has been introduced into TMLI treatment. Its integrated kilovolt FBCT scanner enables accurate evaluation of the difference in dose distribution between plan and delivery.

**Fig. 4:**
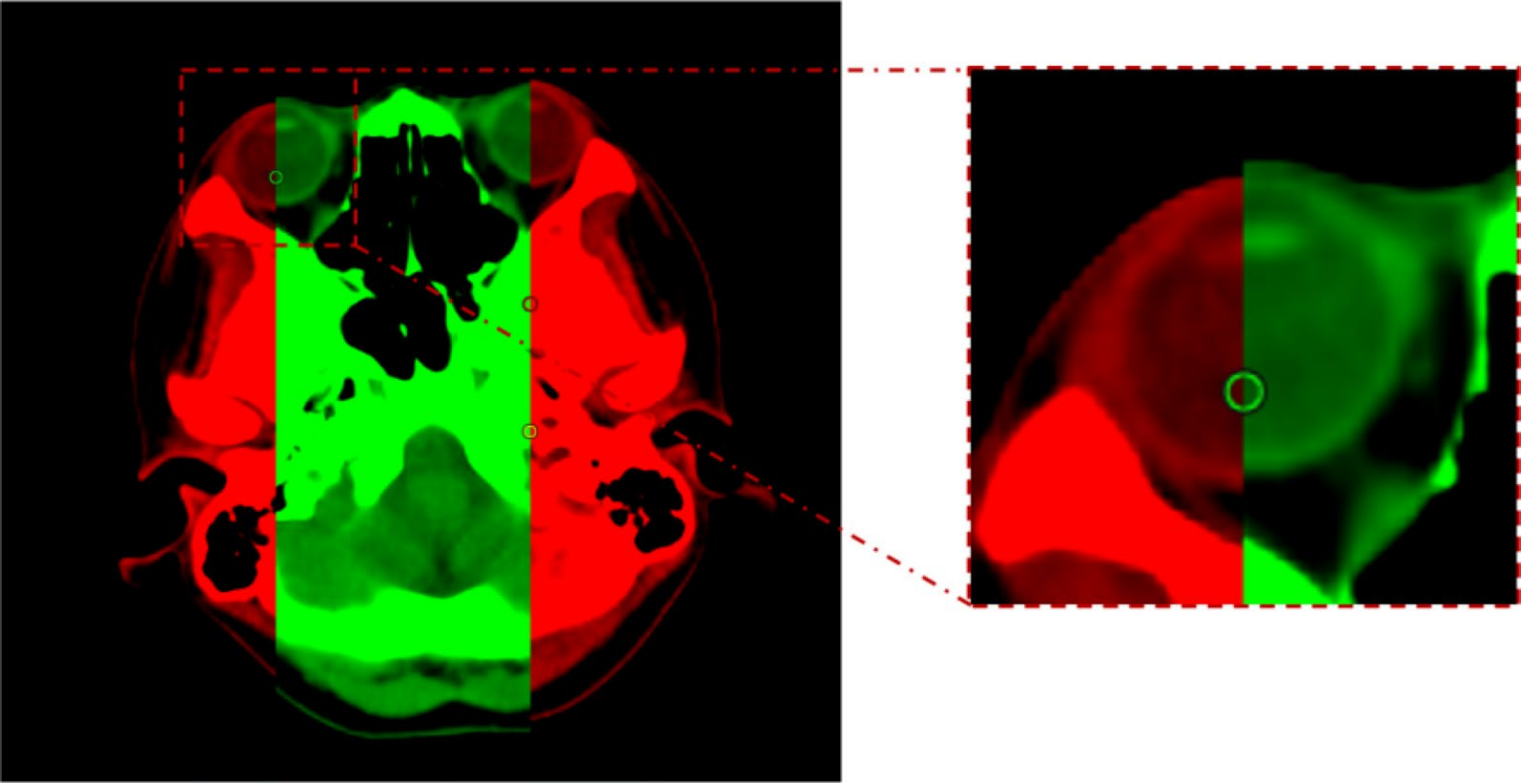
Example of the lens position difference between the simulation CT and the FBCT (obtained in IGRT). The red mask covered the planning CT and the green mask covered the FBCT

In this paper, we firstly demonstrate the technical feasibility and efficiency of uRT-linac 506c linear accelerator for TMLI treatment. The results showed that this CT-linac is able to achieve the adequate dose coverage of PTVs while keeping the acceptable toxicity of crucial organs. The treatment time of it is significantly shorter than the HT, suggesting its efficiency in clinical practice. In our previous study treating 27 patients with HT based TMI/TMLI, average beam-on times of Plan_upper_ and Plan_lower_ are, respectively, 46 min (ranging from 36 to 56 min*)*, 16 min (ranging from 13 to 20 min*)*, and total treatment time is about 2.5 h [9]. Although the patient number used in [9] is different from this study, we can still find that the average beam-on time with uRT-linac 506c, which is a C-arm linear accelerator, is obviously shorter than HT with a difference varying from 16 to 50 min [9. 17–19]. Shahid et al. reported that the total treatment time with Halcyon ring gantry linear accelerator varied from 83 and 91 min [20], which is similar to ours. Instead of using VMAT technique to make the Plan_upper,_ we have chosen dIMRT to save planning time, regarding that the uTPS VMAT allowed only maximal simultaneous optimization of 1440°0(4 full arcs). For the upper body target with VMAT, four sub-plans would need to be made; in addition, the dose gradient matching technique should be applied between adjacent sub-plans. Secondly, we evaluated the difference of planning and re-calculated dose distribution in the TMLI treatment. From the results, we have the following findings: (1) There is no significant difference (*p* > 0.05) between planned dose and re-calculated dose on most of selected organs, except for right lens (*p* < 0.05, D_max_, Wilcoxon signed-rank test). The consistency of planning and re-calculated dose distribution can reflect the good treatment quality to some extent. The actual delivered maximum dose of right lens is obviously larger than the planned dose of it. Figure 4 shows the example of the lens position difference between the simulation CT and the FBCT obtained in IGRT. As can be seen, the volume of lens is very small and the tiny position shift can lead to large offset of lens. This offset caused the dose difference between planning and delivery of lens. (2) There is a significant difference between dose distribution of planning and delivery for PTVs (*p* < 0.05, Wilcoxon signed-rank test). Specifically, the prescription dose of 10 Gy covered 94.79% of the PTV_bone_ for planning, while it covered 92.17% of the PTV_bone_ for delivery. The prescription dose of 12 Gy covered 94.68% of the PTV_lymph_ for planning, while it covered 90.07% of the PTV_lymph_ for delivery. These coverage differences may be caused by the poor repeatability of the patient position, especially for arms and hands, which is the same as the result of scholar Springer [21]. As for the treatment planning quality, for the small bowel, kidneys, lens, liver and lungs dose, our results were obviously lower than that of Fogliata et al. [22] and Wong et al. [23], but for heart value, our result is lower than that of Wong et al. [23] and Wilkie et al. [24], but higher than that of Fogliata et.al [22].

**Fig. 5:**
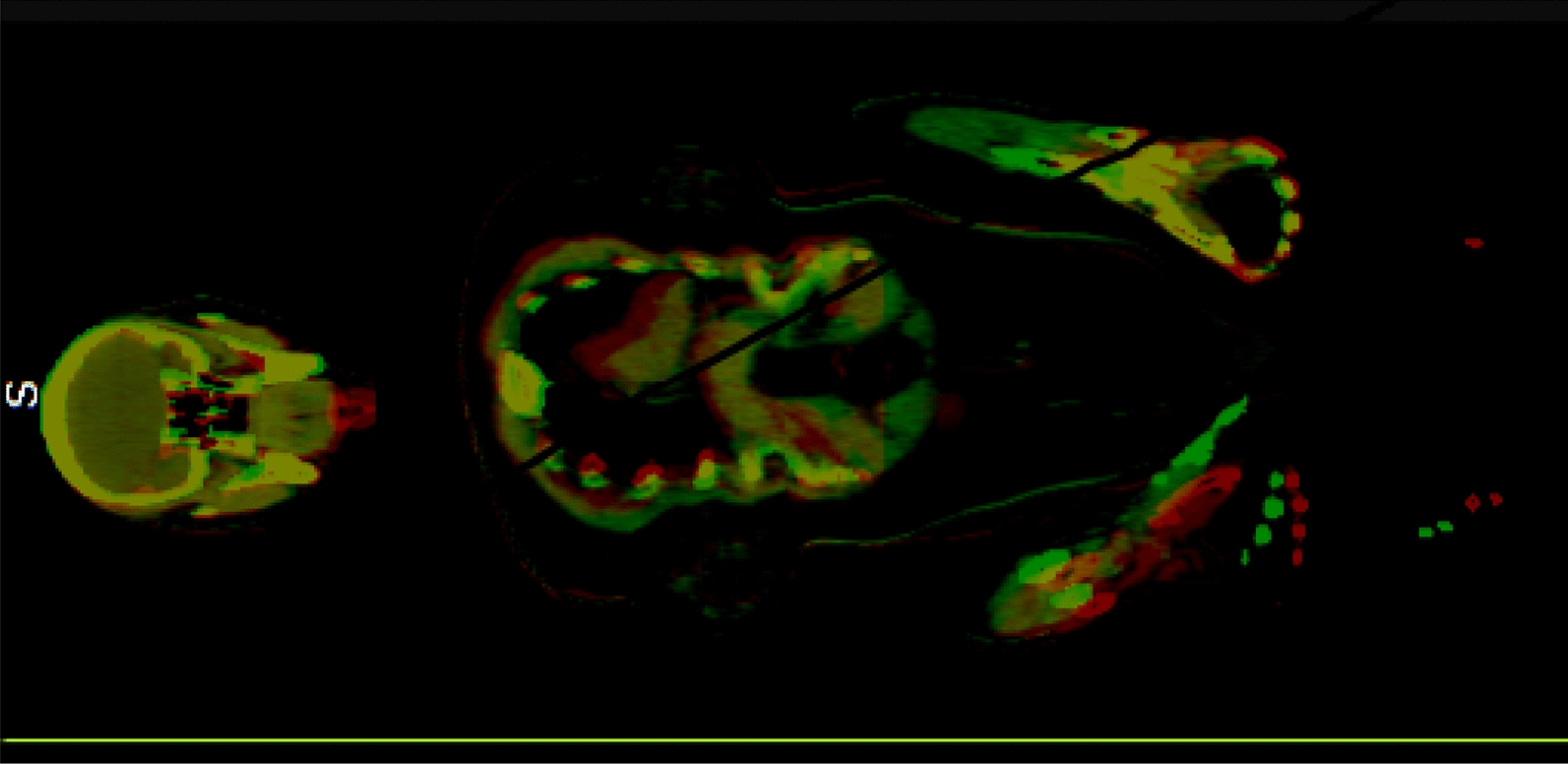
Examples of the arms and hands position difference between the simulation CT and the FBCT (obtained in IGRT). The green mask covered the planning CT and the red mask covered the FBCT. The comparison of the simulation CT and the FBCT

For the patient immobilization, the reproducibility of the arms and hands is severely influenced by the long treatment time. In particular, the patient grasped the self-made fixator with both hands and maintained the same position during the upper body treatment for more than 30 min. This leads to poor reproducibility of the arms and hands. Figure 5 shows the arms and hands position difference between the simulation CT and the FBCT (obtained in IGRT). Although this immobilization method has the mentioned limitation, it can provide a patient with more freedom to seek help in an emergency. During the TMLI treatment, the patients were very weak, and even vomited sometimes. If the head, arms, and hands are fixed by a thermoplastic mask, similar to previous researches [25], when the patients vomit, it is difficult for nurses to notice, and the patients may choke due to vomiting. Considering these situations, we decided to sacrifice a little prescription coverage for patient safety.

Re-calculated dose to the best of our knowledge, this paper is the first attempt to evaluate the dose distribution of delivery. From the results, we can see that poor repeatability of the patient position is still one of the biggest challenges for precision radiotherapy. In the future, some attempts can be made to alleviate this problem. For example, expanding the contour of some small OARs (e.g., lens) for treatment planning.

This study has some limitations, including a small sample size and variations in the types and stages of cancer, as well as the overall health status of the patients. Additionally, there were no follow-up results available to assess the long-term effectiveness of radiation therapy. These factors may impact the generalizability of the findings and limit the ability to draw definitive conclusions about the efficacy of radiation therapy as a cancer treatment option.

## Conclusion

This research first exhibited that the uRT-linac 506c is an efficient device for TMLI treatment. The results showed that uRT-Linac 506c can provide adequate dose coverage for PTVs with clinical acceptable dose of OARs. In addition, the dose difference between planning and delivery was evaluated by comparing the planned dose to the re-calculated dose on the IGRT FBCT, which is important for treatment evaluation and plan improvement for the future studies.

## Data Availability

Please contact author for data requests.
